# Perceptions of a Digital Mental Health Platform Among Participants With Depressive Disorder, Anxiety Disorder, and Other Clinically Diagnosed Mental Disorders in Singapore: Usability and Acceptability Study

**DOI:** 10.2196/42167

**Published:** 2023-03-29

**Authors:** Ye Sheng Phang, Creighton Heaukulani, Wijaya Martanto, Robert Morris, Mian Mian Tong, Roger Ho

**Affiliations:** 1 MOH Office for Healthcare Transformation Singapore Singapore; 2 Yong Loo Lin School of Medicine National University of Singapore Singapore Singapore; 3 Department of Psychological Medicine Yong Loo Lin School of Medicine National University of Singapore Singapore Singapore; 4 Institute for Health Innovation and Technology National University of Singapore Singapore Singapore

**Keywords:** mHealth, mobile health, CBT, cognitive behavioral therapy, cognitive behavioural therapy, iCBT, internet-based cognitive behavioral therapy, usability, Post-Study System Usability Questionnaire, PSSUQ, acceptability, mental health, Singapore, depression disorder, anxiety disorder, mental illness, anxiety, depression, depressive

## Abstract

**Background:**

The website *mindline.sg* is a stress management and coping website that can be accessed anonymously in Singapore for free. Although designed to serve individuals who are well or have mild depression and anxiety symptoms, *mindline.sg* may potentially be used by clinicians as an adjunct therapeutic aid for patients with clinically diagnosed mental disorders.

**Objective:**

This study aims to determine the perceived usability, acceptability, and usefulness of *mindline.sg* among individuals with diagnosed mental disorders in a clinical setting.

**Methods:**

A cross-sectional study with 173 participants was conducted in the waiting room of a psychiatrist’s office at the National University Hospital in Singapore. Participants waiting for an appointment were given 30 minutes and a simple set of instructions to use three features of *mindline.sg*. They subsequently answered a set of web-based survey questions via their smartphones, including a 16-item subset of the Post-Study System Usability Questionnaire (PSSUQ) for usability measurement and 5 questions designed to understand the perceived usefulness and acceptability of *mindline.sg*. Multiple linear regression is used to determine the associated demographic factors with overall PSSUQ score. A chi-square test is performed to investigate associations of psychiatric condition with users’ responses on acceptability and perceived usefulness of *mindline.sg*. For this study, *P*<.05 is considered significant.

**Results:**

We observed that the overall (mean 2.86, SD 1.46), system usefulness (mean 2.74, SD 1.46), and information quality (mean 2.98, SD 1.33) subscores of the PSSUQ survey are within a 99% CI of a literature-derived norm, which all have the interpretation of having high perceived usability. However, interface quality (mean 2.98, SD 1.33) scored lower than the literature-derived norm, although it is still better than the neutral score of 4. We find participants with lower than a General Certificate of Education O-Level or N-Level education tend to give a lower usability score as compared to others (β=.49; *P*=.02). Participants who have not been hospitalized previously due to their condition are also more likely to give a lower PSSUQ score as compared to individuals who have been hospitalized (β=.18; *P*=.03). The platform *mindline.sg* is also deemed to be generally useful and acceptable with all the survey questions receiving more than a 60% positive response. We found no association between the type(s) of self-reported psychiatric disorder(s) and the perceived usefulness and acceptability of *mindline.sg*.

**Conclusions:**

Our results show that *mindline.sg* is generally perceived as usable and acceptable by individuals with a diagnosed mental disorder in Singapore. The study suggests improving usability among individuals with lower education levels. Particularly promising is the finding that previously hospitalized individuals have significantly higher perceived usability and satisfaction of the website, suggesting potential impact could be found among a moderately to severely at-risk clinical population. The effectiveness of *mindline.sg* as an adjunct therapy for individuals with diagnosed mental disorders should therefore be explored in future studies.

## Introduction

According to the United Nations News, mental disorders affect nearly 1 billion people worldwide [1]. There is also a large treatment gap in mental disorders, which is defined as the difference between the numbers of patients needing and receiving mental health treatment. It was estimated that 76%-85% of the people with severe mental disorders receive no medical treatment in low- and middle-income countries, and that number is around 35%-50% in high-income countries [2]. In Singapore, which is considered a high-income country, 78.6% of individuals met the criteria of needing mental health care but did not receive any treatment or help [3].

The low cost and high accessibility of digital therapeutic tools for mental health have the potential to bridge some of the treatment gaps in Singapore. Indeed, some studies have found evidence to support the use of internet-based mental health self-help tools [4-6].

In June 2020, the Ministry of Health Office for Healthcare Transformation, a subsidiary of the Ministry of Health, launched *mindline.sg* [7], an anonymous, digital mental health platform. This platform was developed to empower users in Singapore with knowledge, tools, and pathways to self-care as well as resources to help individuals seek out professional help when needed [8]. The website has since rapidly expanded to now include more than 500 curated resources, a self-assessment tool, and an emotionally intelligent artificial intelligence chatbot from Wysa that deploys a suite of interactive digital therapeutic exercises based on cognitive behavioral therapy. In the 2 years following its launch, *mindline.sg* received over 485,000 unique visitors.

The platform *mindline.sg* was not designed to serve individuals with moderate to severe anxiety or depression or those with clinically diagnosed mental disorders. However, it could eventually be expanded to aid health care professionals as an adjunct to therapy for these individuals. To successfully expand *mindline.sg* to users with diagnosed mental disorders, its usability and acceptability must first be evaluated among this population. Additionally, studies have found that higher acceptability improves uptake and adherence to digital intervention programs [9,10]. This study could also generate insights into product improvement and expansion.

In this study, we aimed to determine the perceived usability and acceptability of *mindline.sg* among patients with diagnosed mental disorders within a clinical setting. The primary objective of the study was to determine the perceived usability of *mindline.sg* through the Post-Study System Usability Questionnaire (PSSUQ). The secondary objective was to determine the perceived usefulness and acceptability of *mindline.sg* through a custom survey.

## Methods

### Study Design and Recruitment

A cross-sectional study with 173 participants was conducted from April 2021 to January 2022 in the waiting room of a psychiatrist’s office at the Department of Psychological Medicine, National University Hospital, Singapore. Participants waiting for an appointment were given 30 minutes and a simple set of instructions to use three prominent features of *mindline.sg*: (1) a novel self-assessment and “wellness triaging” questionnaire consisting of a dynamically evolving set of questions from among the Patient Health Questionaire-9 (PHQ-9) and Generalized Anxiety Disorder-7 (GAD-7) surveys, which aims to help users understand their current levels of anxiety or depression and to direct them to appropriate content; (2) an emotionally intelligent chatbot from Wysa that conducts a range of cognitive behavioral therapy (CBT)–inspired digital therapeutic tools and can also converse with the user in a free form; and (3) a collection of resources provided by health care ecosystem partners on topics such as mental health literacy, employment support, caregiver support, financial support, fitness tips, and domestic abuse support. Following the 30-minute usage period, the participants answered a set of web-based survey questions, including a reduced version of the PSSUQ with 16 questions designed to measure the perceived usability and satisfaction with the platform and 5 yes/no questions designed to understand the acceptability and perceived usefulness of the website.

The participants were recruited by the Department of Psychological Medicine, National University Hospital in Singapore. Participation was optional, and no remuneration was given. Participants between the ages of 21 and 65 years who were waiting for their psychiatrist appointment at the hospital were invited to the study. For safety reasons, we excluded participants with any form of cancer or major neurological disorder (eg, epilepsy and stroke), heart disease (eg, ischemic heart disease), lung disease (eg, chronic obstructive pulmonary disease), liver disease (eg, liver failure), or kidney disease (eg, kidney failure). All participants were required to provide physical informed consent before the commencement of the study.

### Ethics Approval

Ethics approval was granted by the National Healthcare Group Domain Specific Review Board (NHG DSRB 2020/01326) for a study period from January 2021 to January 2022.

### Web-Based Survey Design

The web-based survey consisted of 3 sections. In the first section, participant demographic data were collected. This included age, gender, marital status, education level, annual income, employment status, race, and medical history, as shown in Table 1.

**Table 1:** Demographic data of study participants (N=173).

Demographic information of participants		Values, n (%)
**Gender**		
	Male	66 (38.2)
	Female	107 (61.9)
**Age group (years)**		
	≤20	1 (0.6)
	21-30	83 (48)
	31-40	40 (23.1)
	41-50	35 (20.2)
	>50	14 (8.1)
**Education level**		
	Below GCE^a^ O-Level or N-Level	5 (2.9)
	GCE O-Level or N-Level equivalent	19 (11)
	Diploma, A-Level, or equivalent	66 (38.2)
	Undergraduate degree and above	79 (45.7)
**Marital status**		
	Single	115 (66.5)
	Married	58 (33.5)
**Annual income (SGD)^b^**		
	0	49 (28.3)
	<30,000	46 (26.6)
	30,000-60,000	49 (28.3)
	60,001-100,000	15 (8.7)
	>100,000	14 (8.1)
**Race**		
	Chinese	119 (68.8)
	Malay	24 (13.9)
	Indian	14 (8.1)
	Other	16 (9.3)

^a^GCE: General Certificate of Education.

^b^A currency exchange rate of SGD $1=US $0.74 is applicable.

The second section was modelled after the reduced version of the PSSUQ, which is a widely deployed usability quantification survey [11]. The PSSUQ consists of 16 questions that are scored on a 7-point scale (from 1 as “strongly agree” to 7 as “strongly disagree”; Multimedia Appendix 1). The scores determine an overall satisfaction scale, computed as the average score across all 16 items (and so takes a value between 16 and 112) and 3 subscales of system usefulness, information quality, and interface quality, taking values in the ranges of 6-42, 6-42, and 4-24, respectively, each computed as the average score across various subsets of the items. For all scales, lower scores indicate better usability. The PSSUQ questionnaire has shown a satisfactory level of reliability, sensitivity, and validity [12,13].

The third section of the survey consisted of the following 5 yes/no questions constructed by the study team to measure the acceptability and perceived usefulness of the major features of *mindline.sg*:

1. Did you like taking the “I need help to manage my emotions” questionnaires?
2. Did you find the resources that are listed useful?
3. Did you like talking with the Wysa chatbot (the penguin)?
4. Did you find any of the exercises recommended by *mindline.sg* or Wysa (the emotionally intelligent chatbot) useful?
5. Would you recommend *mindline.sg* to a friend?

These questions have not been tested for reliability, sensitivity, and validity.

### Statistical Analysis

We used multiple linear regression analyses to discover factors that are associated with the overall PSSUQ scale, such as age, marital status, annual income, education level, and medical history. The empirical distribution of the overall PSSUQ scale was not well modelled by a Gaussian distribution (Shapiro-Wilks test: *P*<.001), so a power transformation was applied to the PSSUQ scales before training.

To investigate any relationship between the self-reported psychiatric conditions and the responses to the 5 yes/no survey questions measuring the acceptability and perceived usefulness of the platform, we performed chi-square tests based on self-reported psychiatric conditions. Three population comparisons were performed: (1) between participants with and without depressive disorder, (2) between participants with and without anxiety disorder, and (3) between participants diagnosed with any other psychiatric conditions and those diagnosed with either depressive or anxiety disorders. We noted that 10 participants indicated they either did not have any psychiatric disorder or did not know their diagnosis; the responses of these participants were excluded from this analysis.

All statistical tests were 2-tailed. We reported factors at *P<*.05 as significant, and corrections for multiple comparisons were not used.

## Results

### Demographic Data

The demographic data collected, summarized in Table 1, revealed the majority of the participants were female (107/173, 61.9%), between the ages of 21 and 30 years (83/173, 48%), had an education level of “undergraduate degree and above” (79/173, 45.7%), and were single (115/173, 66.5%).

### Medical History

The medical history data are summarized in Table 2. The majority of the participants (88/173, 50.8%) self-reported being diagnosed with depressive disorder, were most likely to be on either medication (84/173, 48.6%) or both medication and psychotherapy or counselling (71/173, 41%), and had not been hospitalized due to their psychiatric condition (107/173, 61.9%).

**Table 2:** Data on the medical history of the participants.

Medical history			Values, n (%)
**Do you suffer from any psychiatric condition?**			
	Depressive disorder	88 (50.8)	
	Anxiety disorder	38 (22)	
	Both anxiety and depressive disorder	14 (8.1)	
	Others (eg, bipolar disorder, attention deficit hyperactivity disorder, adjustment disorder, schizophrenia, borderline personality disorder, and alcoholism)	23 (13.3)	
	Unknown or undiagnosed	10 (5.8)	
**How long (in years) have you suffered from this psychiatric condition(s)?^a^**			
	<1	29 (17.8)	
	≥1 to <5	44 (27)	
	≥5 to <10	40 (24.6)	
	≥10 to <15	27 (16.6)	
	>15	23 (14.1)	
**What kind of treatment are you receiving for this psychiatric condition(s)?^a^**			
	Medication	84 (48.6)	
	Psychotherapy or counseling	2 (1.2)	
	Both medication and psychotherapy or counselling	71 (41)	
	Others	6 (3.5)	
	Not under any treatment	10 (5.8)	
**Have you been hospitalized due to this psychiatric condition(s)?^a^**			
	Yes	66 (38.2)	
	No	107 (61.9)	
**Have you been on depression medication?^a^**			
	Yes	133 (77.3)	
	No	40 (23.1)	
**Do you suffer from any other chronic medical conditions?^a^**			
	Yes	48 (27.8)	
	No	125 (72.3)	

^a^Unknown or undiagnosed participants are not required to answer this question.

### PSSUQ Results

In Table 3, we report the mean and SD values of the PSSUQ overall score and subscores. A meta-analysis of 5 years of usability studies (which were predominantly on speech recognition systems, though the meta-analysis showed a good ability to generalize) provided the means and 99% CIs of analyzed PSSUQ scores (Table 3) [11]. We will henceforth refer to the literature-derived mean and 99% CIs as *PSSUQ norms*. A lower score indicates better usability.

In Table 4, we report the parameter estimates in a multiple linear regression model (a Box-Cox transformation) of the PSSUQ overall satisfaction scale onto the participant demographic and medical history data.

**Table 3:** The overall Post-Study System Usability Questionnaire (PSSUQ) scores and subscores as well as the norms.

Questions	PSSUQ score, mean (SD)	PSSUQ norms, mean (99% CI) [10]
System usefulness (questions 1-6)	2.74 (1.46)	2.80 (2.57-3.02)
Information quality (questions 7-12)	2.98 (1.33)	3.02 (2.79-3.24)
Interface quality (questions 13-15)	2.82 (1.59)	2.49 (2.28-2.71)
Overall (questions 1-16)	2.86 (1.46)	2.82 (2.62-3.02)

**Table 4:** Parameter estimates in multiple linear regression (a power transformation) of the overall satisfaction with the Post-Study System Usability Questionnaire (PSSUQ) scale onto the participant demographic and medical history data. Italicized *P* values are significant.

Factors associated with the overall satisfaction PSSUQ score		β	*P* value
Age		–.00	.76
Gender (male vs female)		–.00	>.99
Marital status (married vs single)		.155	.13
**Annual income (vs no income; SGD)^a^**			
	>30,000	.175	.09
	30,000-60,000	.201	.06
	60,001-100,000	.205	.23
	>100,000	.132	.45
**Education level (vs university degree and above)**			
	Below GCE^b^ O-Level or N-Level certification or equivalent	.491	*.02*
	GCE O-Level or N-Level certification or equivalent	.044	.75
	Diploma, A-Level, or equivalent	.053	.58
**Medical condition (vs others)**			
	Depressive disorder	.137	.13
	Anxiety disorder	–.063	.51
Have not been previously hospitalized for a psychiatric condition (vs having been previously hospitalized)		.177	*.03*

^a^A currency exchange rate of SGD $1=US $0.74 is applicable.

^b^GCE: General Certificate of Education.

### Acceptability and Perceived Usefulness of *“mindline.sg”*

The last section of the survey consists of five yes/no questions designed to measure the acceptability and perceived usefulness of *mindline.sg*. The overall responses were largely positive with all the 5 questions receiving more than a 60% positive response. The question “Did you find the resources that are listed useful?” received the highest percentage of positive responses (86.7% of the users). The question “Did you find any of the exercises recommended by *mindline.sg* or Wysa useful?” received the lowest percentage of positive response (60%). Table 5 shows the full results of the survey. We found no significant differences between the responses of any subgroups.

**Table 5:** An analysis comparing the distributions of the responses to the survey questions measuring the usefulness and acceptability of the platform between participants segmented into three groups: (1) participants with depressive disorder (DD) versus participants without DD; (2) participants with anxiety disorder (AD) versus participants without AD; and (3) participants with other psychiatric conditions, excluding DD and AD, versus participants with DD and AD. The *P* value of chi-square tests comparing the various subpopulation distributions is reported in the final column. There are no differences in survey responses between the diagnoses at significance level (*P*=.05).

Items		Total (N=163), n (%)	DD vs no DD				AD vs no AD				Others vs DD and AD			
			DD (n=102), n (%)	No DD (n=61), n (%)	Chi-square (df)	*P* value	AD (n=52), n (%)	No AD (n=111), n (%)	Chi-square (df)	*P* value	Others (n=23), n (%)	DD and AD (n=140), n (%)	Chi-square (df)	*P* value
**Did you like taking the “I need help to manage my emotions” questionnaires?**														
	No	31 (19.7)	22 (21.6)	9 (14.8)	0.8 (1)	.39	11 (21.2)	20 (18)	0.1 (1)	.79	2 (8.7)	29 (20.7)	1.2 (1)	.28
	Yes	132 (80.4)	80 (78.4)	52 (85.2)	—^a^	—	41 (78.8)	91 (81)	—	—	21 (91.3)	111 (79.3)	—	—
**Did you find the resources that are listed useful?**														
	No	23 (13.3)	14 (13.7)	9 (14.6)	0.0 (1)	.96	8 (15.4)	15 (13.5)	0.0 (1)	.94	3 (13)	20 (14.3)	0.0 (1)	.87
	Yes	140 (86.7)	88 (86.3)	52 (85.2)	—	—	44 (84.6)	96 (86.5)	—	—	20 (86)	120 (85.7)	—	—
**Did you like talking with the Wysa chatbot (the penguin)?**														
	No	53 (31.8)	37 (36.3)	16 (26.2)	1.3 (1)	.25	14 (26.9)	39 (35.1)	0.7 (1)	.39	6 (26)	47 (33.6)	0.2 (1)	.64
	Yes	110 (68.2)	65 (63.7)	45 (73.8)	—	—	38 (73)	72 (64.9)	—	—	17 (73.9)	93 (66.4)	—	—
**Did you find any of the exercises recommended by *mindline.sg* or Wysa useful?**														
	No	63 (39.9)	45 (44.1)	18 (29.5)	2.8 (1)	.092	19 (36.5)	44 (39.6)	0.0 (1)	.84	8 (34.8)	55 (39.3)	0.0 (1)	.86
	Yes	100 (60.1)	57 (55.9)	43 (70.5)	—	—	33 (63.5)	67 (60.4)	—	—	15 (65.2)	85 (60.7)	—	—
**Would you recommend *mindline.sg* to a friend?**														
	No	40 (24.3)	29 (28.4)	11 (18)	1.7 (1)	.19	11 (21.2)	29 (26.1)	0.2 (1)	.62	5 (21.7)	35 (25)	0.0 (1)	.94
	Yes	123 (75.7)	73 (71.6)	50 (81)	—	—	41 (78.8)	82 (73.9)	—	—	18 (78.3)	105 (75)	—	—

^a^Not applicable.

## Discussion

### Usability Findings

Comparing the PSSUQ overall score (describing the perceived usability of *mindline.sg* among the survey respondents) to the literature-derived norms (Table 3), we found that the system usefulness (mean 2.74, SD 1.46), information quality (mean 2.98, SD 1.33), and the platform overall score (mean 2.86, SD 1.46) were perceived as “good” and were comparable to most other digital apps (within a 99% CI of the literature-derived norm). Although the interface quality score (mean 2.98, SD 1.33) is lower in this regard than most other digital apps, it is also perceived as “good” because it is above the neutral score of 4 on the PSSUQ scale.

Based on the results of the multiple linear regression with the overall PSSUQ scores, we found that education level is the factor with the highest association with the PSSUQ score (the largest magnitude coefficient is reported in Table 4). In particular, participants with an education lower than an O-Level or N-Level General Certificate of Education tend to give *mindline.sg* a lower usability score (β=0.49; *P*=.02). Because the majority of the resources on *mindline.sg* are text-based and require a certain level of English literacy to use, we find the results in line with our expectations. However, these findings are in contrast with earlier studies, which found a weak to no correlation between education level and usability of connected medical devices and internet-based CBT (iCBT) platforms [10,13].

In addition, participants who have not been previously hospitalized due to their psychiatric condition are likely to give a poorer overall PSSUQ score compared to participants who have been hospitalized (β=0.177; *P*=.03). At the point of writing, we could not find any prior study to explain this finding.

### Acceptability and Perceived Usefulness Findings

We found that *mindline.sg* is generally acceptable to participants with self-reported mental disorders, with all 5 questions having more than 60% positive response. These results are consistent with the findings from a meta-analysis that found iCBT platforms were acceptable and effective for patients with depression and anxiety disorders [14].

When comparing patients’ responses on *mindline.sg*, acceptability and perceived usefulness between the three different mental condition subgroups (as illustrated in Table 5), we found no significant differences between responses by type of psychiatric disorder.

### Limitations

The participants were given only 30 minutes to use the three features of *mindline.sg* before they were asked to complete the survey, but some of the therapeutic exercises provided by the Wysa chatbot on the website could take around 20-30 minutes to complete. This could explain why the questions on “Did you find any of the exercises recommended by *mindline.sg* or Wysa useful?” received the least amount of positive response (60%) compared to the other acceptability questions, as the participants may not have had enough time to fully explore these exercises. Other resources are usually completed in a shorter amount of time, as they take the form of articles and videos that generally take less than 10 minutes to consume. The short usage period of *mindline.sg* in this study may affect the generalizability of this study.

Additionally, the medical history collected in this survey is self-reported and has not been independently verified with clinical records (this was not put forward to the ethics committee to protect patient confidentiality and preserve the anonymity feature of the *mindline.sg* platform). The data reported in our study are also from participants who had an appointment in the Department of Psychological Medicine in National University Hospital and were not randomly selected, which could result in some form of selection bias. Given the limitations mentioned above, any generalization from this study should be evaluated with caution.

Although the PSSUQ norms were used as a basis for comparison with our collected results, it is important to note that the norms were established by products from a variety of sources (which were predominantly speech recognition systems) and at different stages of development [12].

Although we compare our results of acceptability to a meta-analysis, it is also important to note that many of the studies in the meta-analysis use adherence and patient satisfaction in a longer-term treatment program as a proxy for acceptability [13]. Since our participants only use *mindline.sg* for around 30 minutes, the answer to the 5 questions is instead used as a proxy to acceptability.

Lastly, as the evaluation of usability in mobile health varies substantially [15], it presents a challenge for us to compare our findings to previously published usability results. Although the PSSUQ questionnaire has shown a satisfactory level of reliability, sensitivity, and validity [11,12], the 5 yes/no questions that were constructed by the study team as a proxy for acceptability have not been tested for validity and reliability. The nature of the yes/no questions could also limit the range of responses as compared to a Likert-type scale.

### Conclusions

Despite the limitations mentioned above, this study shows that *mindline.sg* could be a viable self-help tool for individuals with diagnosed mental health conditions due to its well-rated usability and acceptability. Furthermore, the accessibility of a free, anonymous, and web-based tool like *mindline.sg* allows people with diagnosed mental conditions to access these services at any time and from the comfort and privacy of their homes. However, the clinical effectiveness of *mindline.sg* as a mental health resource for people diagnosed with mental conditions has not yet been validated and might be an important focus for future studies.

## Appendix

Multimedia Appendix 1 Poststudy usability questionnaire used for this study.

## Abbreviations

CBT: cognitive behavioral therapy
GAD-7: Generalized Anxiety Disorder-7
iCBT: internet-based cognitive behavioral therapy
MOH: Ministry of Health
PHQ-9: Patient Health Questionaire-9
PSSUQ: Post-Study System Usability Questionnaire
