# Supplementary material for: Perceptions of a Digital Mental Health Platform Among Participants With Depressive Disorder, Anxiety Disorder, and Other Clinically Diagnosed Mental Disorders in Singapore: Usability and Acceptability Study
Source: JMIR Hum Factors. 2023 Mar 29;10:e42167. doi: 10.2196/42167 (PMC10132018; doi:10.2196/42167)
Supplement: Multimedia Appendix 1 [file humanfactors_v10i1e42167_app1.docx]

**The Post-Study Usability Questionnaire Version 3 (for *mindline.sg*)**

**Strongly disagree**

**Strongly agree**

|  |  |  | **1** | **2** | **3** | **4** | **5** | **6** | **7** |
| --- | --- | --- | --- | --- | --- | --- | --- | --- | --- |
| **1** | Overall, I am satisfied with how easy it is to use *mindline.sg*. |  |  |  |  |  |  |  |  |
| **2** | It was simple to use *mindline.sg*. |  |  |  |  |  |  |  |  |
| **3** | I was able to complete the tasks and scenarios quickly using *mindline.sg*. |  |  |  |  |  |  |  |  |
| **4** | I felt comfortable using *mindline.sg*. |  |  |  |  |  |  |  |  |
| **5** | It was easy to learn to use *mindline.sg*. |  |  |  |  |  |  |  |  |
| **6** | I believe I could become productive quickly using *mindline.sg*. |  |  |  |  |  |  |  |  |
| **7** | mindline.sg gave error messages that clearly told me how to fix the problems. |  |  |  |  |  |  |  |  |
| **8** | Whenever I made a mistake using *mindline.sg*, I could recover easily and quickly. |  |  |  |  |  |  |  |  |
| **9** | The information (such as online help, on-screen messages and other documentation) provided with *mindline.sg* was clear. |  |  |  |  |  |  |  |  |
| **10** | It was easy to find the information I needed. |  |  |  |  |  |  |  |  |
| **11** | The information was effective in helping me complete the tasks and scenarios. |  |  |  |  |  |  |  |  |
| **12** | The organization of information on *mindline.sg* screens was clear. |  |  |  |  |  |  |  |  |
| **13** | The interface of *mindline.sg* was pleasant. |  |  |  |  |  |  |  |  |
| **14** | I liked using the interface of *mindline.sg*. |  |  |  |  |  |  |  |  |
| **15** | *mindline.sg* has all the functions and capabilities I expected it to have. |  |  |  |  |  |  |  |  |
| **16** | Overall, I am satisfied with *mindline.sg*. |  |  |  |  |  |  |  |  |
